# Advances in the Renin-Angiotensin-Aldosterone System: Relevance to Diabetic Nephropathy

**DOI:** 10.1100/tsw.2008.69

**Published:** 2008-04-20

**Authors:** Audrey Koitka, Christos Tikellis

**Affiliations:** JDRF Danielle Alberti Memorial Centre for Diabetic Complications, Diabetes and Metabolism Division, Baker Heart Research Institut, Melbourne, Victoria, Australia

**Keywords:** angiotensin II, angiotensin type 1 and type 2 receptors, ACE, ACE2, renin, aldosterone, diabetic nephropathy

## Abstract

Hypertension is now recognized as a key contributory factor to the development and progression of kidney disease in both type 1 and type 2 diabetes. The renin angiotensin system (RAS) and its effector molecule angiotensin II, in particular, have a range of hemodynamic and nonhemodynamic effects that contribute not only to the development of hypertension, but also to renal disease. As a result, therapeutic inhibition of the RAS with angiotensin-converting enzyme inhibitors and/or selective angiotensin II type 1 receptor blockers has been proposed as a key strategy for reducing kidney damage beyond the expected effects one would observe with blood pressure reduction per se. Although the relationship between the RAS and the progression of diabetic renal disease has been known for many decades, recent advances have revealed a more complex paradigm with the discovery of a number of new components. Thus, further understanding of these new components of the renin angiotensin aldosterone system (RAAS), such as the angiotensin type 2 receptor subtype, angiotensin converting enzyme 2, and the recently cloned renin receptor, is likely to have therapeutic implications for disorders such as diabetic nephropathy, where interruption of the RAAS is widely used.

## INTRODUCTION

Diabetic nephropathy (DN) is a microvascular complication of diabetes. Specifically, it represents a major cause of morbidity and mortality in type 1 and type 2 diabetic subjects, and has become the leading cause of end-stage renal disease in the Western world[1]. The renin angiotensin system (RAS) and its effector molecule angiotensin II (AII), in particular, have a range of hemodynamic and nonhemodynamic effects that contribute not only to the development of hypertension, but also to renal disease. As a result, pharmacological inhibition of the RAS has been proposed as a key strategy for reducing kidney damage beyond the predicted effects as a result of blood pressure reduction. Pharmacological agents include angiotensin-converting enzyme inhibitors (ACEi), that block the conversion of the prohormone angiotensin I to the active hormone AII, and selective AII type 1 receptor blockers (ARBs), which competitively inhibit the action of AII at the AT1 receptor subtype[2,3,4,5]. Both compounds are now widely used for reducing renal complications in patients with type 1 and type 2 diabetes. Recently, clinical studies suggested that optimization of blockade of the RAS with a combination of ACEi and ARBs may confer superior renoprotection than either agent as a monotherapy[6,7]. Nevertheless, this dual blockade of the RAS still fails to prevent DN totally[8]. This manuscript reviews recent advances in our understanding of the renin angiotensin aldosterone system (RAAS) and the role of this hormonal cascade in diabetic kidney complications. Furthermore, the discovery of these new components of the RAAS could explain the suboptimal renoprotection seen with current RAS inhibition.

## ROLE OF AII IN THE DEVELOPMENT OF DN

The classical RAS consists of enzymatic pathways leading to the production of AII, which mediates most of its effects via binding to G-protein coupled angiotensin type 1 (AT1) receptors. In the kidney, AII is produced in relatively high levels, and binding to AT1 receptors (present in nephron segments, interstitial cells, and the vasculature) stimulates vasoconstriction, sodium and water retention, and cell growth responses.

Studies on the role of RAS in the pathophysiology of DN mainly focused on the systemic RAS and provided controversial results, with the RAS reported as up-regulated, unchanged, or suppressed in the diabetic kidney[9]. It has become increasingly appreciated that there is a local intrarenal RAS, which acts independently of the systemic RAS and appears to be activated in both human[10] and experimental[11] diabetes.

Overall, these findings suggest a role for AII as a central mediator of renal damage in diabetes. This concept is further supported by several observations. First, AII infusion leads to functional and structural abnormalities, which resemble DN. Second, AII infusion increases albumin excretion rate and plasma creatinine, reduces creatinine clearance, and increases glomerular basement membrane width in streptozotocin-induced diabetic rats[12]. This link between the RAS and glomerular basement membrane width was also suggested in early studies where treatment with the ACEi enalapril reduced glomerular basement membrane width in hypertensive diabetic rats[13].

AII is more than just a vasoconstrictor and has trophic effects independent of its hemodynamic actions. These include direct effects on profibrotic and proinflammatory pathways, including activation of nuclear factor-kappa B (NFkB), stimulation of profibrotic cytokines and their receptors including transforming growth factor-beta (TGFβ) and connective tissue growth factor (CTGF), activation of angiogenic cytokines and related molecules, and promotion of macrophage recruitment and infiltration, partly via increasing expression of the chemokine MCP-1 (monocyte chemotactic protein-1)[14].

### AII and Mesangial Cells

In mesangial cells, AII induces hypertrophy and hyperplasia, and promotes extracellular matrix accumulation by both increasing the synthesis and decreasing the degradation of extracellular matrix components. For example, AII induces the expression of TGFβ1, fibronectin, collagen type 1[15], and CTGF in mesangial cells. Furthermore, AII decreases collagenase (MMP-2) levels and stimulates TGFβ1 secretion in mesangial cells. Finally, AII induces the production of cytokines, chemokines, and growth factors, such as interleukin-6, MCP-1[16], and vascular endothelial growth factor (VEGF)[17], which may, in turn, amplify the renal damage that is occurring in diabetes.

### AII and Glomerular Epithelial Cells (Podocyte)

In podocytes, AII exerts effects that may be relevant to the alteration of the permselectivity of the glomerular filtration barrier. For example, exposure of podocytes to AII results in a redistribution and loss of nephrin, which is associated with changes in cytoskeleton distribution, including loss in stress fibers, cortical accumulation of F-actin, and cell retraction[18]. In addition, AII induces reorganization of F-actin fibers and redistribution of zonula occludens-1 (ZO-1), and these effects are paralleled by increased albumin permeability across podocyte monolayers[19]. In transgenic rats, AT1 receptor overexpression in podocytes leads to protein leakage and structural podocyte damage, progressing to focal segmental glomerulosclerosis[20]. Finally, in podocytes, AII stimulates production of alpha3(IV) collagen protein via mechanisms involving TGFβ and VEGF signaling[21], and promotes both hypertrophy and apoptosis. These widespread effects of AII on various aspects of podocyte biology emphasize the potential role of interrupting the RAS in attenuating or reversing diabetes-related changes in podocyte structure and function.

### AII and Tubular Cells

In tubular cells, AII induces cell hypertrophy[22], apoptosis, and epithelial-myofibroblast transdifferentiation[23]. Furthermore, AII promotes extracellular matrix accumulation both by stimulating the expression of extracellular matrix components[22] and by up-regulating TGFβ receptor type II expression, thus amplifying the effects of TGFβ1 on this particular cell type.

## TREATMENT TARGETS FOR DN: RAS BLOCKADE

Activation of the RAS and the subsequent generation of AII appear to be among the most important mediators of hemodynamic and nonhemodynamic changes in DN, as outlined in detail previously. As a result, blockade of AII formation or action by ACEi or specific antagonists to the AT1 receptor has been proposed as a therapeutic strategy for reducing kidney damage as a result of diabetes. National and international guidelines advocate the use of these agents as first-line therapy to reduce proteinuria and retard the progression of renal disease[24].

Indeed, current data suggest that antihypertensive treatments that block the RAS are particularly effective in reducing renal complications in patients with both type 1 and type 2 diabetes.

### RAS Blockade in Type 1 Diabetes

A landmark study conducted by the Collaborative Study Group in 1993 enrolled 409 type 1 diabetic patients with overt nephropathy (AER >500 mg/24 h), randomizing them to receive captopril or placebo[3]. ACEi therapy was associated with a 48% reduction in the risk of doubling serum creatinine. A difference in blood pressure between the two groups of 2–4 mmHg in favor of ACEi was observed. However, after adjustment for the difference in blood pressure, ACEi still resulted in a significant risk reduction of 43%, suggesting a powerful nonhemodynamic effect of this specific treatment. Furthermore, the ACEi were associated with a 50% reduction in the combined end points of death, dialysis, and transplantation. In a meta-analysis of 12 trials, including microalbuminuric nonhypertensive patients with type 1 diabetes, the risk of progression to macroalbuminuria was reduced by 60% following ACEi initiation with the chance of regression to normoalbuminuria also significantly increased[25].

ARBs have been trialed in patients with type 1 diabetes and nephropathy. Losartan reduced albuminuria by as much as 44% in patients with macroalbuminuria[26]. However, there currently are no studies in type 1 diabetes demonstrating definitive renal outcomes comparable to the study by the Collaborative Study Group using captopril. Given the abundant data supporting the use of ACEi in type 1 diabetic subjects with nephropathy, major guidelines continue to recommend ACEi as first-line therapy.

## RAS Blockade in Type 2 Diabetes

The benefits of RAS blockade above other antihypertensive agents in patients with type 2 diabetes and overt nephropathy or microalbuminuria were more controversial until the publication of three seminal studies in this area: the IDNT (Irbesartan Diabetic Nephropathy Trial)[4], the RENAAL (Reduction of Endpoints in Non-Insulin-Dependent Diabetes Mellitus with the Angiotensin II Antagonist Losartan)[2], and the IRMA-2 (Irbesartan Microalbuminuria)[5].

The BENEDICT (BErgamo NEphrologic DIabetes Complications Trial) study demonstrated that blockade of the RAS with the ACEi trandolapril prevented the progression to microalbuminuria in hypertensive, type 2 diabetic patients with a normal urinary albumin excretion rate[27]. Similarly, ramipril reduced the incidence of microalbuminuria in normoalbuminuric patients with type 2 diabetes from the micro-HOPE study[28]. In a series of relatively underpowered small studies in type 2 diabetic patients with overt nephropathy, ACEi were shown to be effective, but not significantly better than other treatments, such as β-blockers or nondihydropyridine calcium channel blockers[29,30,31].

Clinical studies with ARBs therapy in type 2 diabetes have been more promising in terms of demonstrating possible superiority above other antihypertensive drug classes. Several studies demonstrated the effects of ARBs on the reduction of albuminuria[8,32] and the progression of renal disease from microalbuminuria to macroalbuminuria. Losartan, valsartan, candesartan, telmisartan[33], and irbesartan[5] all demonstrated antiproteinuric effects in clinical trials. Several studies have now demonstrated hard clinic end point benefits of ARBs above other antihypertensive agents.

Nevertheless, very few studies have compared the efficacy of these classes when investigating the relative renal benefits in diabetic patients.

Experimental mouse and rat models of diabetes have explored the comparative benefits of ACEi and ARBs. These experiments demonstrated similar effects on blood pressure, urinary albumin excretion, glomerular ultrastructure, and glomerular filtration rate[34]. More recently, several small, relatively independent, clinical studies compared ARBs to ACEi. In one study, losartan was compared to enalapril in 103 patients with type 2 diabetes, hypertension, and microalbuminuria. There were no differences in blood pressure or albuminuria after 1 year of active treatment[35]. The more recent Diabetics Exposed to Telmisartan and Enalapril (DETAIL) study was a noninferiority study conducted in 250 diabetic, hypertensive patients with microalbuminuria (80%) or overt albuminuria (20%). After a follow-up of 5 years, there was no statistical difference in decline in GFR or albuminuria between the two groups[33]. Thus, there do not appear to be significant differences between ACEi and ARBs in type 2 diabetic patients with nephropathy based on a small number of comparison studies. However, international and national recommendations favor ARBs in type 2 diabetic subjects, based on clinical trials, such as IDNT and RENAAL, demonstrating reductions in hard clinical end points, such as doubling of serum creatinine and progression to end-stage renal disease[2,4].

Unfortunately, patients with type 2 diabetes are unlikely to achieve the recommended goal blood pressure of <130 mmHg systolic and 80 mmHg diastolic[36] utilizing a single antihypertensive agent. Therefore, combination therapies utilizing both ACEi and ARBs may be necessary[2,4] and are described below.

## Combination Therapy

### *ACEi and ARB Agents*

There has been increasing interest regarding the combination of ACEi and ARB based on the hypothesis that this dual blockade of the RAS could be more complete than for either agent alone[6]. Experimental studies in diabetic and hypertensive rats, administering combination therapy including ACEi and ARB, were more effective at reducing blood pressure, urine albumin excretion, and glomerulosclerosis than monotherapy with either class of agent[37].

Several clinical studies explored the possible benefits of dual RAS blockade. The Candesartan and Lisinopril Microalbuminuria (CALM) study examined 197 type 2 diabetic hypertensive patients with microalbuminuria. Dual therapy reduced albumin excretion to a greater degree, which was only statistically significant when compared to ARB monotherapy. Dual therapy significantly reduced blood pressure compared to either agent alone[8]. Several further small studies involving type 2 diabetic subjects with hypertension and albuminuria demonstrated significant reductions in albuminuria with combination therapy when compared to single agents[7].

The true renal benefits of dual RAS blockade with ACEi and ARB require ongoing evaluation with larger randomized control trials. Indeed, in nondiabetic renal disease, dual blockade was reported in the COOPERATE study to reduce the risk of an important end-point, end-stage renal disease[38]. Ongoing studies, such as ONTARGET[39], which will assess the combination of the ACEi ramipril and the ARB telmisartan, although focusing on renal end points and including nondiabetic subjects, may provide further additional information in this area.

### *ACEi and NEP Agents*

Neutral endopeptidase (NEP) is a zinc metallopeptidase that constitutes the major enzymatic pathway for the degradation of natriuretic peptides and a secondary pathway for the degradation of kinins. The combined blockade of NEP and ACE leads to increased local levels of natriuretic peptides, bradykinin, and nitric oxide, as well as reduced levels of AII, thereby enhancing vasodilation, reducing peripheral vascular resistance and blood pressure, and improving blood flow[40]. Previous studies suggested a superior renoprotective effect of dual ACEi/NEP inhibition, including omapatrilat, compared with ACE inhibition in experimental diabetic nephropathy[41,42,43]. In a model of accelerated renal injury, omapatrilat treatment in diabetic apolipoprotein E-knockout mice was associated with superior renoprotective actions over those of the ACEi quinapril[44]. In another model of progressive renal disease, the subtotal nephrectomy model, omapatrilat had a more potent antihypertensive effect compared with the ACEi fosinopril, and also conferred superior renoprotection[45]. However, with major safety consensus with respect to omapatrilat including angioedema[41], the clinical role of these dual ACE/NEP inhibitors is not currently under intensive investigation.

## NEW AND FUTURE POTENTIAL AGENTS TO TARGET DN: THE INCREASING COMPLEXITY OF THE RAAS

Over the last decade, most interest was focused on AII and the blockade of its function and/or action via ACEi and/or ARBs. However, this classical model of the RAS focusing on the vasoconstrictor AII has been replaced by a more complex paradigm with the discovery of a number of new components (Fig. 1). Thus, the renin angiotensin aldosterone system (RAAS) may now be seen as a balanced equilibrium between opposing forces, vasodilatation and vasoconstriction, natriuresis and salt retention, angiogenesis and apoptosis.

### Aldosterone Antagonists

Aldosterone has been suggested to play a role in the progression of DN. Clinical studies have demonstrated a relationship between increased levels of aldosterone and deterioration in renal function. It is likely that aldosterone, independently of the RAS, is an important pathogenic factor in progressive kidney disease, promoting fibrosis and target organ dysfunction[46].

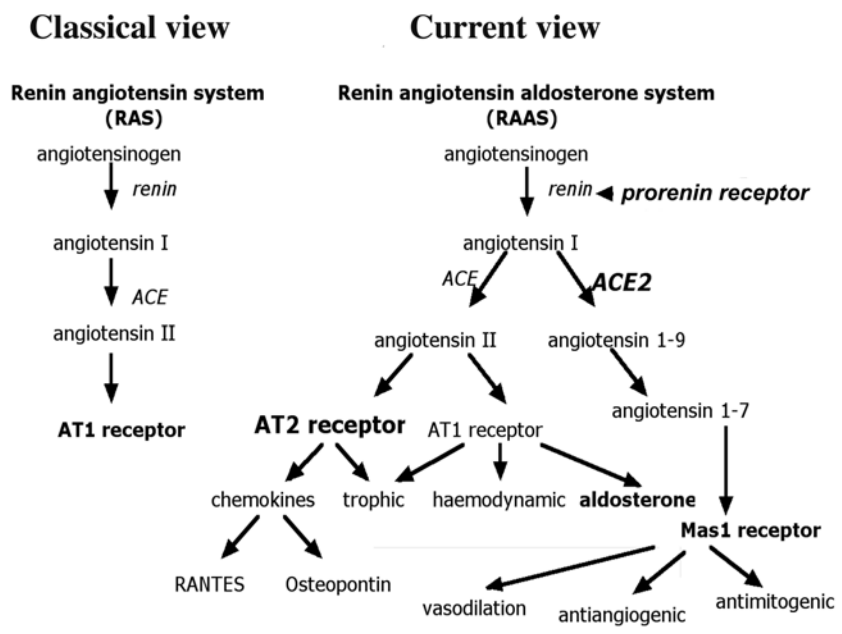

**FIGURE 1.** The evolving view of the RAS.

The role of aldosterone antagonists in renal disease was examined initially using a relatively nonselective inhibitor, spironolactone. Several experimental studies reported that blockade of the RAAS with spironolactone reduced some features of diabetic renal injury in streptozotocin-treated rats, specifically renal fibrosis, renal macrophage infiltration, and renal elevations in TGFβ gene expression[47,48]. A more recent study showed that the use of a more selective mineralocorticoid receptor antagonist, eplerenone, reduced albuminuria, glomerular hypertrophy, early mesangial matrix expansion, and osteopontin expression in both streptozotocin-treated uninephrectomized rats and *db/db* mice[49]. In a study using eplerenone in hypertensive, microalbuminuric type 2 diabetic patients, proteinuria was reduced to a similar extent to that seen with ACE inhibition[46]. Thus, eplerenone is considered a potentially useful therapy for preventing progression of DN, but its exact role in diabetic renal disease remains to be fully defined[50].

## AT2

AII binds with similar affinity to two major receptor subtypes: the type 1 (AT1) and the type 2 (AT2) receptors[51]. While most of the well-known effects of AII are mediated via the AT1 receptor, there is growing interest in effects mediated via the AT2 receptor. The AT2 receptor is ubiquitously expressed in fetal tissues, but its expression declines after birth[52]. In adults, AT2 receptor expression is detectable in the pancreas, heart, vasculature, adrenals, brain, and kidney[51,53,54]. The AT2 receptor is overexpressed in pathologic situations involving tissue remodeling or inflammation, including kidney damage[55,56,57] and specifically the diabetic kidney (Fig. 2). However, the role of the AT2 receptor in renal disease is not completely understood. It has been postulated that the AT1 and AT2 receptors have opposing actions on proliferation and apoptosis. The proliferative properties of AII were considered to be associated with the AT1 receptor, whereas the AT2 receptor was viewed to promote apoptosis, cell growth inhibition, renal nitric oxide production, and glomerular monocyte infiltration[58]. However, the effects of AT2 receptor activation are not always uniform and reproducible, and there is currently no consensus on the role of the AT2 receptor in renal damage. Recently, it has emerged that blockade of the AT2 receptor may confer a degree of renal protection in certain settings[56,59,60]. The AT2 receptor may participate in the

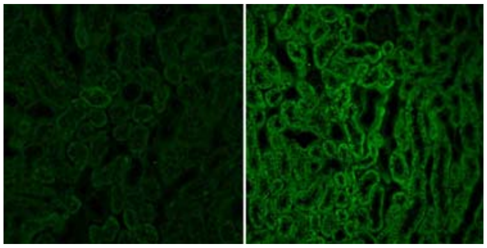

**FIGURE 2.** Representative microscopic pictures of immunohistochemical staining for the AT2 receptor in paraffin kidney sections in control (left) and diabetic (right) C57Bl6 mice. Magnification, ×200.

development of kidney damage via the generation of reactive oxygen species. Moreover, it was shown that the blockade of both AT1 and AT2 receptors is necessary to stop completely the inflammatory process in a model of renal injury[60]. In addition, the AT2 receptor was shown to promote activation of the key proinflammatory signaling mediators, such as NFkB[60,61]. Thus, further investigation of the role of the AT2 receptor subtype specifically in DN is necessary, particularly in view of the importance of the RAAS and the widespread use of RAAS inhibitors in clinical practice.

## ACE2

ACE2 was recently identified as a novel enzyme involved in the regulation of the RAAS[62,63]. ACE2 leads to the generation of angiotensins such as A1-7 (Fig. 1), a peptide with antiangiogenic and vasodilatory actions that antagonize the effects of AII[64]. Increasingly, this enzyme is being considered as part of a counter-regulatory mechanism to the classical RAAS pathway[65].

Although less widely expressed than the ubiquitous enzyme ACE, ACE2 is expressed in kidney, heart, and testis[62,63], suggesting that, like ACE, this novel enzyme is involved in cardiovascular and renal homeostasis. The full-length cDNA coding for human ACE2 predicts a protein of 805 amino acids having 41% homology with the N-terminal catalytic domain of ACE, and a hydrophobic region near the C-terminus likely to serve as a membrane anchor. Thus, like ACE itself, ACE2 is predicted to have the topology of a type 1 membrane protein with the catalytic domain on the extracellular surface.

ACE2 may function to limit the vasoconstrictor action of AII not only by acting to inactivate this potent vasoconstrictor, but also by promoting the formation of a counteracting peptide, A1-7. Thus, the identification and characterization of ACE2 has uncovered an exciting new area of renal physiology as well as providing a possible novel therapeutic target[66]. Indeed, a recent study showed that ACE2 mRNA is reduced in animal models of hypertension and ACE2 knockout mice exhibit cardiac contractile dysfunction, which could be restored by concomitant ACE ablation[67]. Since the discovery and characterization of ACE2, there has been increasing interest in peptides, which are either cleaved or generated by this enzyme. The recently discovered putative A1-7 receptor Mas-1 adds further complexity to the RAAS, particularly within the kidney[68]. Preliminary studies by our group have localized this receptor to a subpopulation of renal tubules and pilot experiments suggest that this receptor is down-regulated in the diabetic kidney, but up-regulated by ACE inhibition.

Immunohistochemical studies by our group[69] and others[62] show that in the kidney, both ACE2 and ACE protein are localized predominantly to epithelial cells of the distal tubule. We have already reported that ACE2 protein expression is reduced in the diabetic kidney (Fig. 3) and this reduction is prevented by ACEi therapy[69]. Given our findings of altered expression levels of ACE2 in the diseased

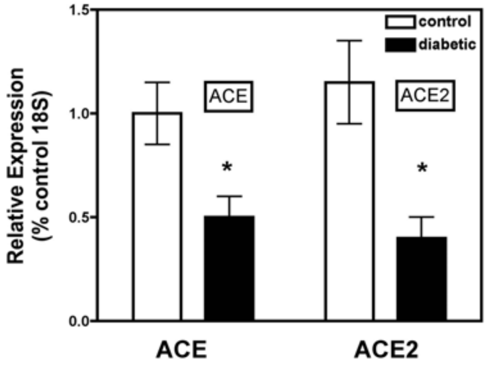

**FIGURE 3**. Expression of ACE and ACE2 mRNA in control and diabetic rodent tubules (*$p$ < 0.05 vs. controls).

kidney and heart, we postulate a critical role for ACE2 in angiotensin metabolism, and predict changes in both expression and distribution of ACE2 in diseases associated with excessive or deficient AII.

## Renin Receptor

The existence of renin binding proteins has been known for sometime, although the exact nature was unknown. Recently, a prorenin binding protein or prorenin receptor was cloned[70] and its role is currently being clarified[71]. This receptor, known as N14F, is a 45-kD, 350-amino-acid protein with no homology to any other known protein[70]. Receptor expression at the gene level was detected in the heart, brain, and placenta, and to a lesser extent in the kidney. Two major roles have been identified at this stage, which could be relevant to diabetic complications. First, this receptor appears to be responsible for cellular effects of renin, independent of the generation of angiotensin. This is particularly relevant in diabetes where increased levels, particularly of prorenin, which also binds to this receptor, were observed in plasma from diabetic patients at risk of or with nephropathy (Fig. 4)[72]. This view that prorenin receptor is a direct enhancer of disease in diabetes has been controversial since no cellular mechanisms involving a receptor and postreceptor signaling had previously been identified.

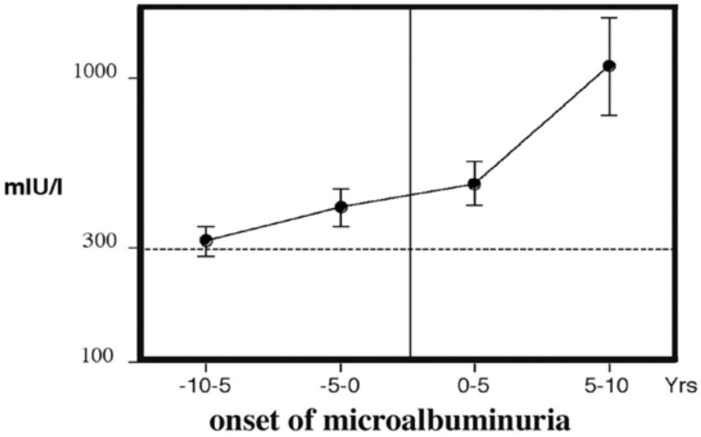

**FIGURE 4.** Plasma prorenin (log scale, y axis) in type I diabetic patients before and after the development of microalbuminuria. Adapted from Allen et al.[72].

Second, it was shown recently that inhibiting prorenin receptor activation by infusion of a “handle region protein” (HRP), functioning as a decoy, effectively prevented streptozotocin-diabetes-induced glomerulosclerosis[73]. Although the relevance of these findings in human DN remains to be established, it is likely that blockade of prorenin receptor *via* a HRP may lead to new treatments for DN, particularly in the context of concomitant hypertension.

Although these discoveries focus on prorenin, renin is also a relevant target for diabetic complications. Indeed, the renin inhibitior aliskiren was tested clinically and was shown to lower blood pressure in hypertensive patients with or without type 2 diabetes[74,75], and is also effective in combination with a thiazide diuretic, an ACEi, or an ARB[76,77]. Preliminary evidence suggests that aliskerin has beneficial effects on DN, as it was shown to be renoprotective in a rodent model of diabetes[78]. Also, in a small trial of patients with type 2 diabetes, aliskerin was shown to lower albuminuria as well as blood pressure significantly[79]. However, these findings need to be confirmed in larger clinical trials.

## CONCLUSION

Over the last 20 years, great progress has been made in defining the cellular and molecular mechanisms implicated in the development and progression of DN. This has led to more rational treatments and, in particular, the widespread use of agents that interrupt the RAAS. Over the next few years, newer agents are being trialed in the diabetic population and this hopefully will lead to a further improvement in the prognosis of DN. Furthermore, with new discoveries in the field of RAAS, it is likely that additional agents and therapeutic strategies will be developed, leading to optimization of renoprotective regimens in diabetes.

## ACKNOWLEDGMENTS

Dr. Koitka is supported by the Association de Langue Française pour l'Etude du Diabète et des Maladies Métaboliques (ALFEDIAM), as well as being supported by an international National Institutes of Health (NIH) grant (RO1HL083452). Dr. Tikellis is supported by a JDRF advanced postdoctoral fellowship.
